# Supplementary figures and images for: Trickle infection and immunity to Trichuris muris
Source: PLoS Pathog. 2019 Nov 15;15(11):e1007926. doi: 10.1371/journal.ppat.1007926 (PMC6881069; doi:10.1371/journal.ppat.1007926)

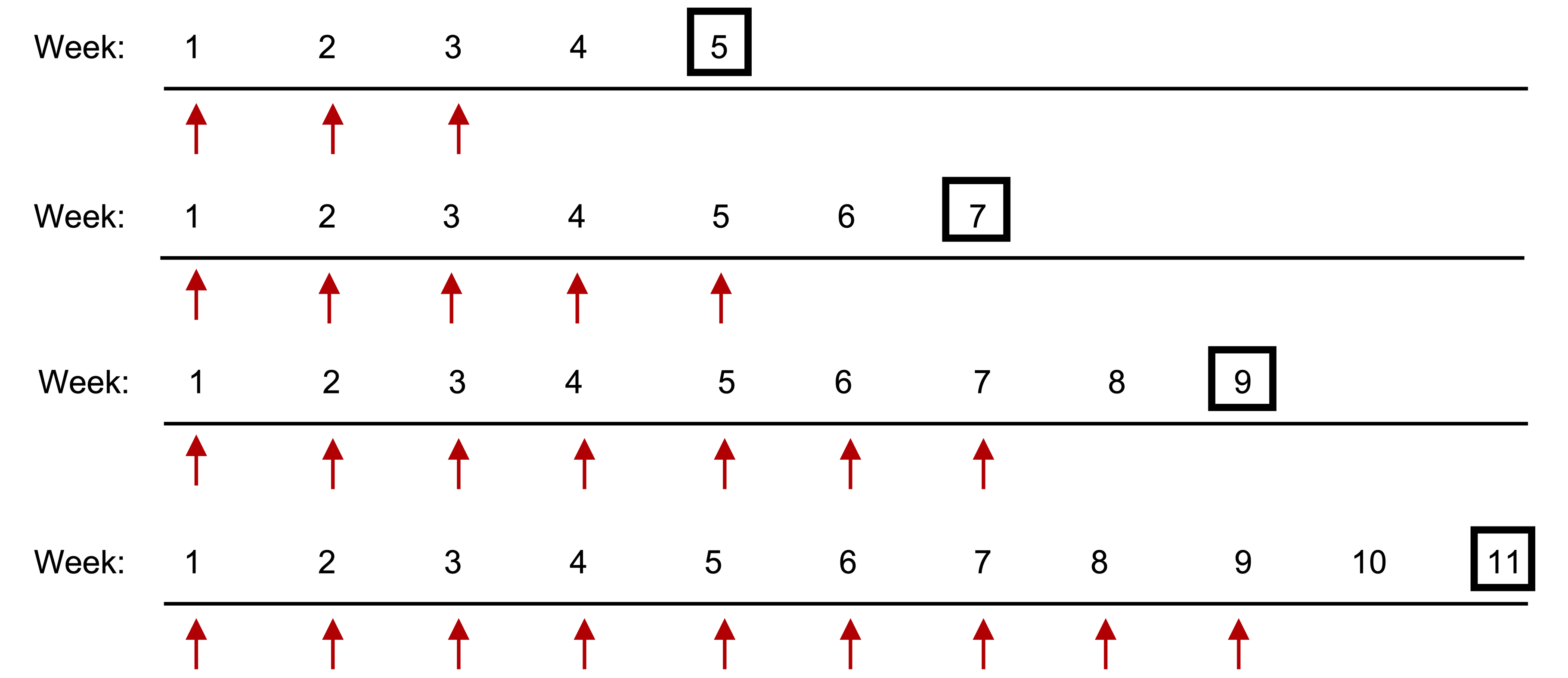

Supplement: S1 Fig — Schematic representing a trickle infection regime. C57BL/6 mice were infected weekly with low doses of T. muris (20 eggs) for 3, 5, 7 or 9 weeks (red arrow). 2 weeks after the final infectious dose at week 5, 7, 9 and 11, worm burdens and immune responses were analysed (black box). (TIF) [file ppat.1007926.s001.tif]

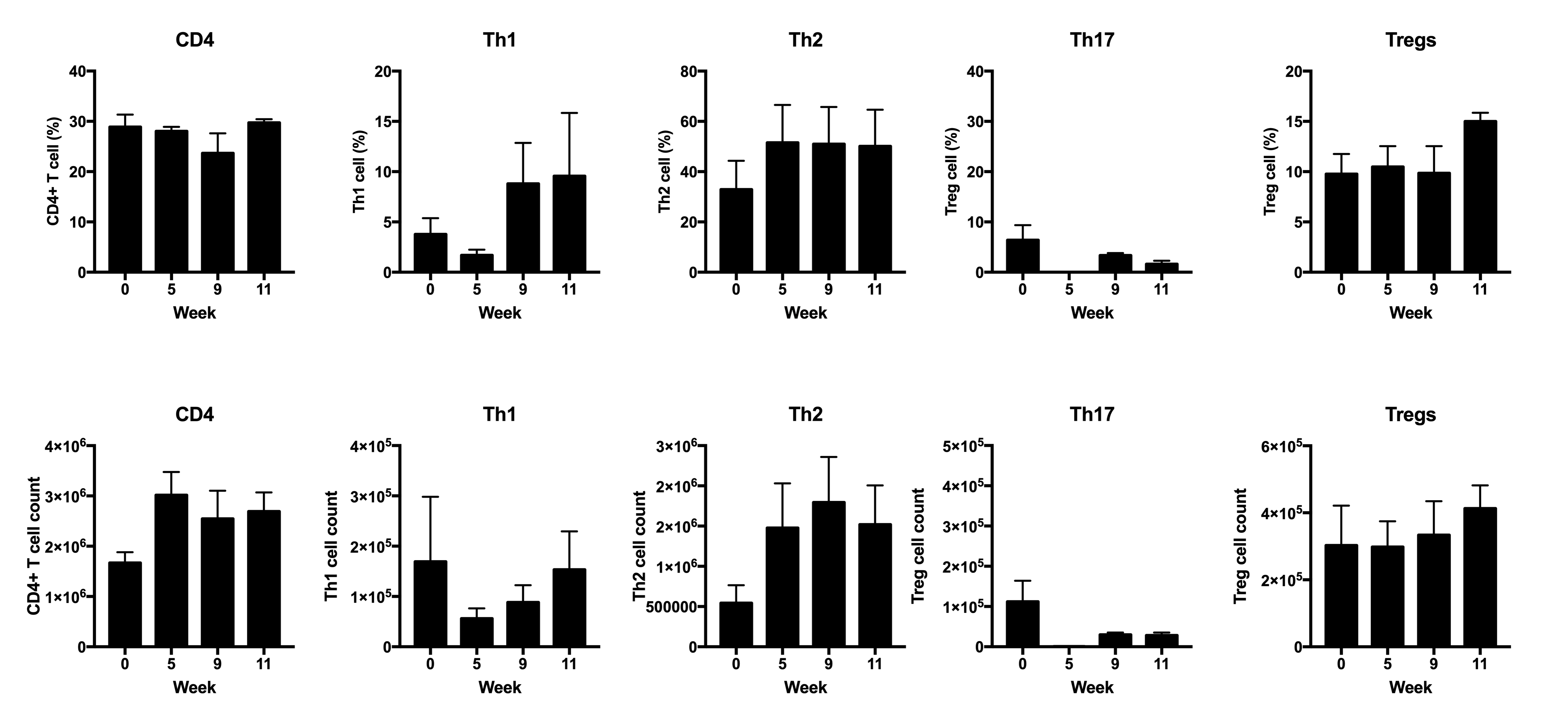

Supplement: S2 Fig — C57BL/6 mice were infected weekly with low doses of T. muris and 2 weeks after the final trickle infection CD4+ T cells in the MLN were analyzed by FACs. CD4+ cell subsets were identified by transcription factor expression; Th1 (Tbet+), Th2 (GATA3+), Th17 (RORγt+), Tregs (FOXP3+). n = 10, from two independent experiments. CD4+ T cell percentage calculated as percentage of all live cells. T cell subset percentage calculated as percentage of all CD4+ cells. (TIF) [file ppat.1007926.s002.tif]

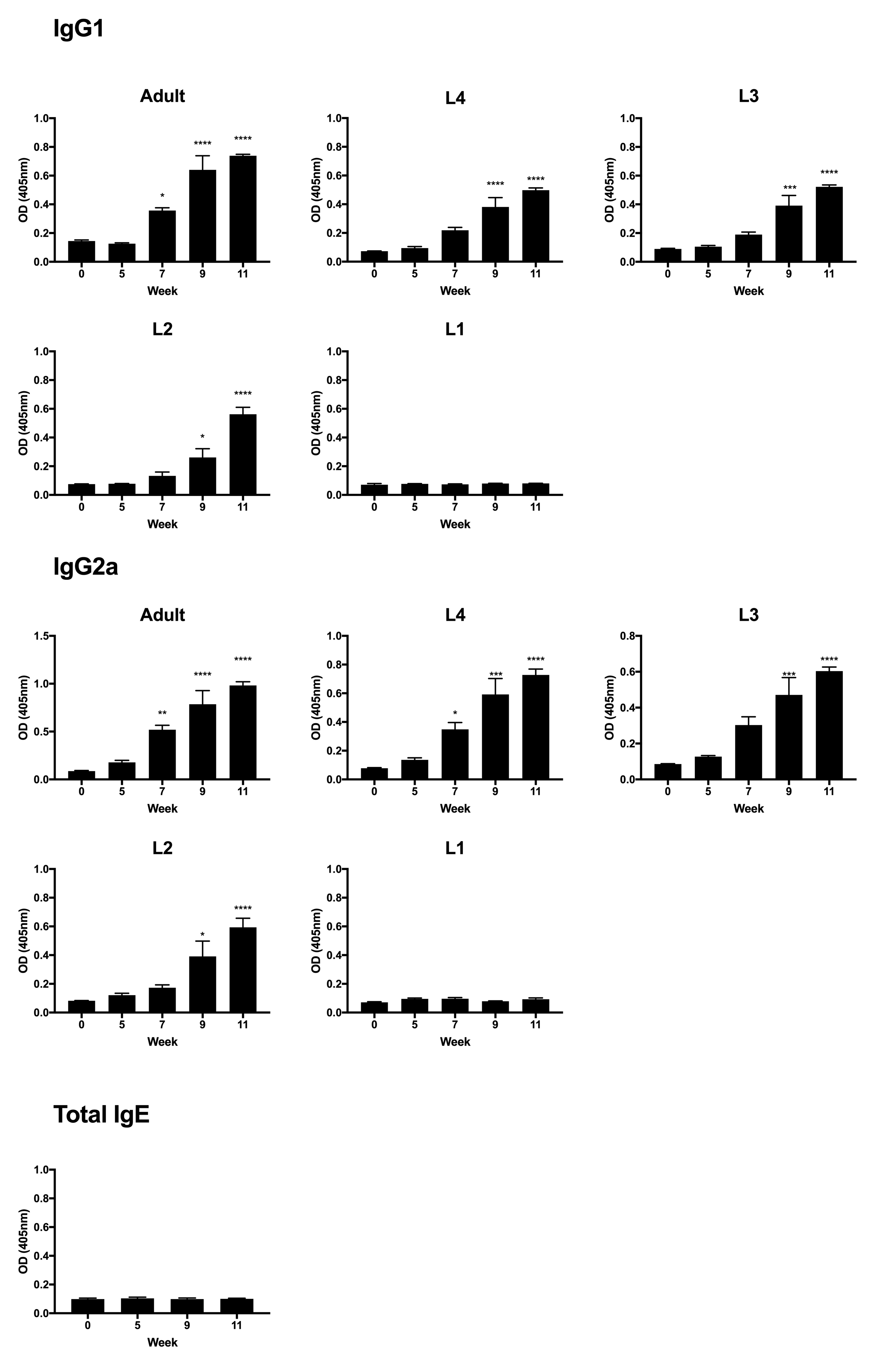

Supplement: S3 Fig — Antibody responses measured from sera collected from trickle infected mice. Antibody levels were measured using indirect ELISA where serially diluted serum from individual mice was incubated in 96-well plates coated with T. muris E/S, then targeted with antibodies against mouse IgG1 or IgG2a/c. Values are given as arbitrary optical density values of the substrate measured at 405 nm. The antibody response specific for adult worms and larval stages 1–4 was measured. (A) IgG1 response. (B) IgG2a/c response. (C) Total IgE response. n = 5, statistical analysis completed by a one way-ANOVA. Data presented as mean +/- SEM, * = p<0.05, ** = p<0.01, **** = p< 0.0001. (TIF) [file ppat.1007926.s003.tif]

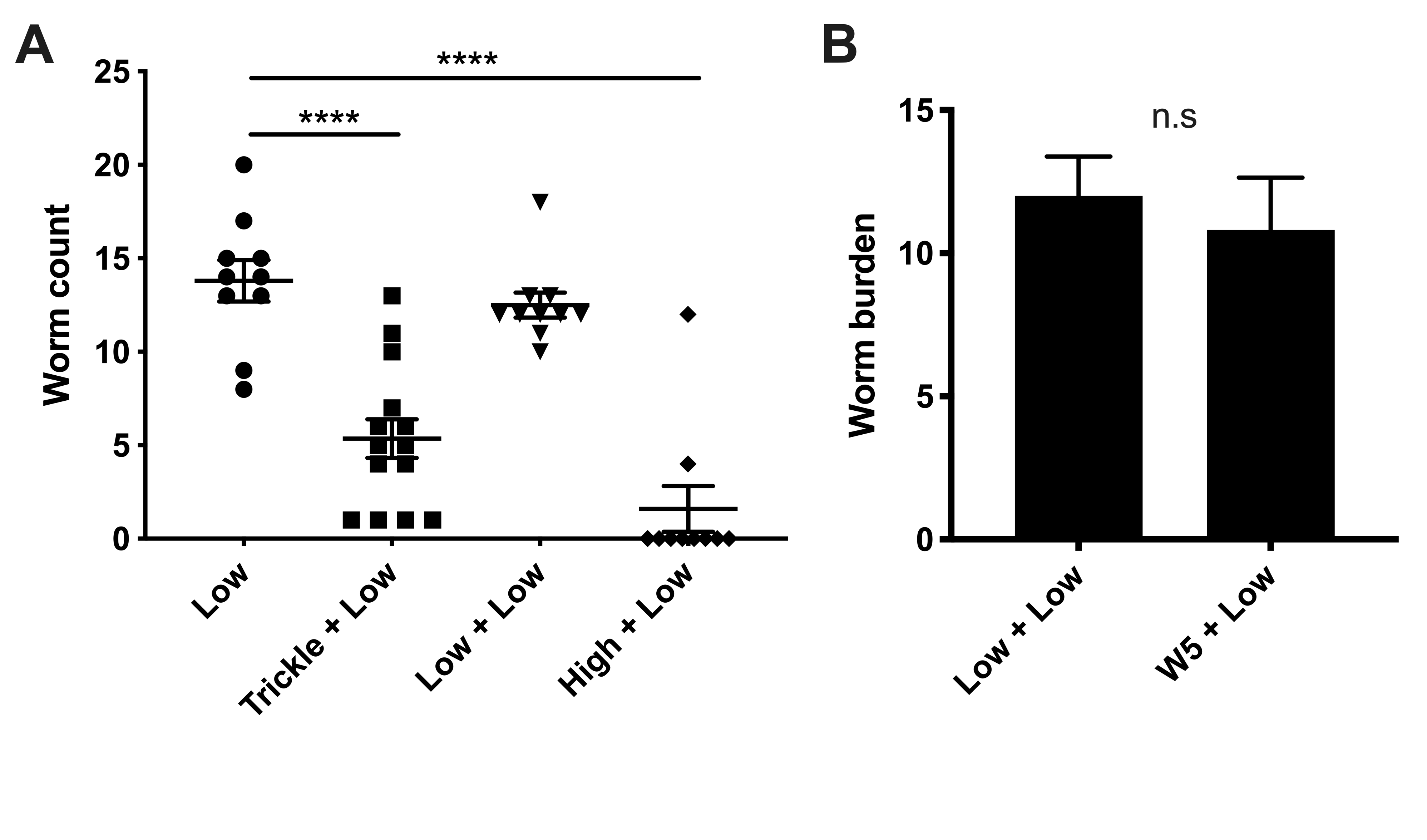

Supplement: S4 Fig — To determine whether trickle infection could protect against a challenge infection, trickle infected mice were either left to expel all worms naturally or worms were removed by anti-helminthic treatment. (A) At week 30, following either a single high or low dose infection or trickle of low dose infections, when no worms were present, determined by measuring faecal egg output, mice were challenged with a single low dose infection. Control mice received a low dose challenge at week 30 post infection n = 10 or greater. (B) Following a single low dose infection or a trickle of 3 low dose infections, mice were treated with anti-helminthics to remove final worms at week 11 post infection. Worm expulsion was confirmed by the absence of eggs in the faeces, Mice were then challenged with a low dose infection one week following anti-helminthic treatment. Worm burden was assessed by eye under dissecting microscope. n = 5 representative of two independent experiments, statistical analysis completed by a one way ANOVA or an unpaired t test. Data presented as mean +/- SEM, * = p<0.05, ** = p<0.01, *** = p<0.001, **** = p< 0.0001. (TIF) [file ppat.1007926.s004.tif]

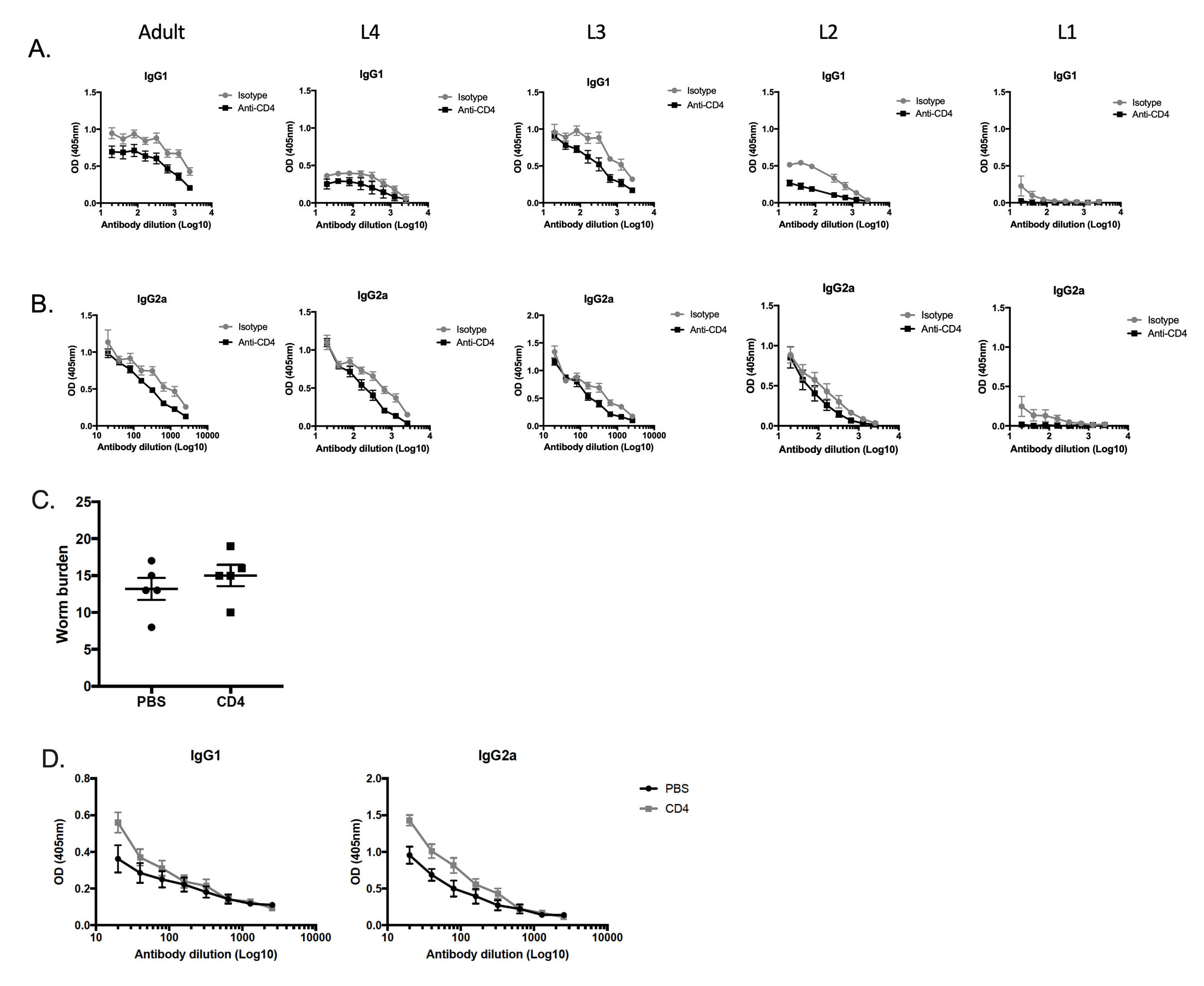

Supplement: S5 Fig — Sera from T. muris infected mice depleted of CD4+ T cells was collected and IgG1 and IgG2a responses specific for T. muris larval stages were quantified. Antibody levels were measured using indirect ELISA where serially diluted serum from individual mice was incubated in 96-well plates coated with T. muris E/S, then targeted with antibodies for against mouse IgG1 or IgG2a/c. Values are given as arbitrary optical density values of the substrate measured at 405 nm. A) IgG1 response to adult worms and larval stages 1–4. B) IgG2a response to adult worms and larval stages 1–4. Isotype control in grey. Anti-CD4 treatment mice in black n = 5. (C-D) CD4+ T cells were isolated and purified from week 11 trickled infected mice. 2x10^6^ CD4+ T cells were injected i.v. into C57BL/6 mice which then received a single low dose infection (20 eggs) the following day. C) Worm burden was counted at day 35 p.i. D) ELISA to quantify T. muris specific IgG1 and IgG2c levels. Data presented as mean +/- SEM, n = 5. (TIF) [file ppat.1007926.s005.tif]

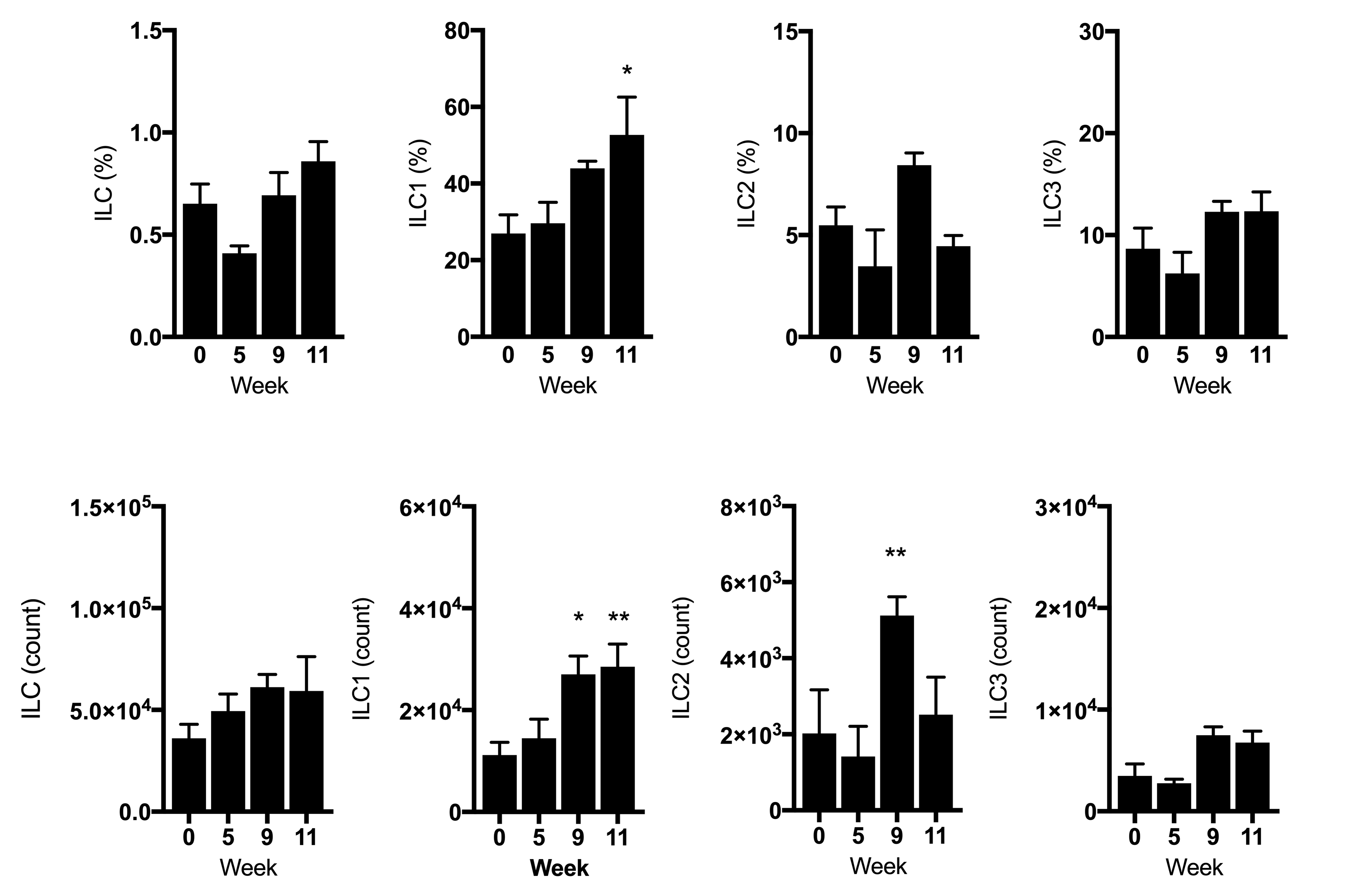

Supplement: S6 Fig — Innate lymphoid cell counts and percentage in the MLN following T. muris trickle infection measured by FACS, identified as lineage negative, CD90.2+, CD127+. Total ILC percentage calculated as percentage of all live cells. ILC subset calculated as the percentage of total ILCs. n = 3, statistical analysis completed by a one way-ANOVA. Data presented as mean +/- SEM, * = p<0.05, ** = p<0.01 (TIF) [file ppat.1007926.s006.tif]

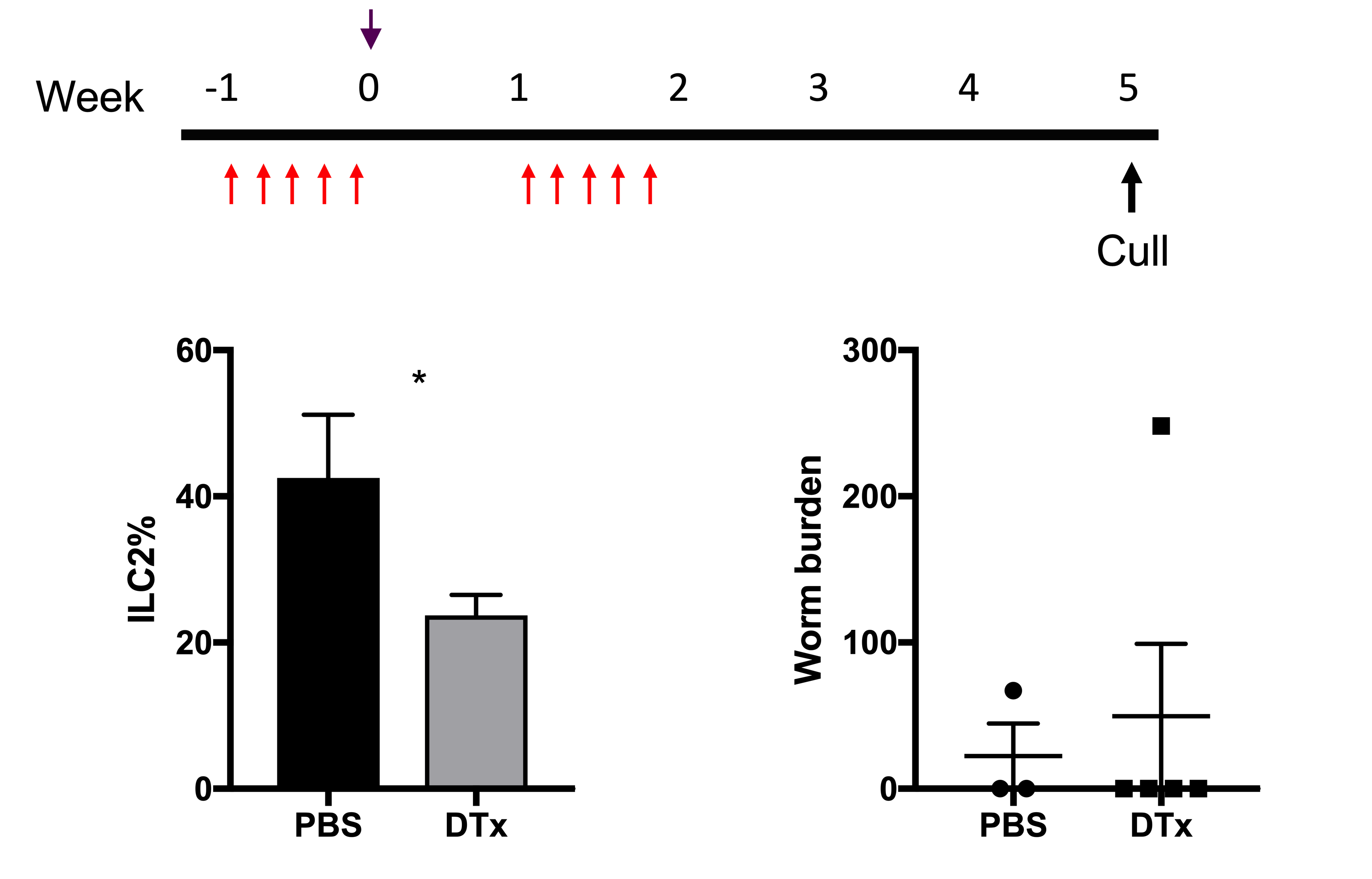

Supplement: S7 Fig — ILC2s were depleted from ICOS-T mice by DTx treatment. (A) Mice received 750ng DTx (red arrow) for 5 days before T. muris high dose (400 eggs) infection (purple arrow) and 1 week after T. muris infection for 5 days. Control mice received PBS control injections at the same time points. Worm burden was analysed at week 5 p.i. (black arrow). (B) Flow cytometry to confirm depletion, ILC2s identified as Lineage-, CD127+, CD90.2+, GATA3+ cells. ILC2% of all ILCs and ILC2 counts. (C) Worm burden of T. muris at day 35 p.i. Statistical analysis completed by an unpaired t-test. Data presented as mean +/- SEM, * = p<0.05. n = 3–5. (TIF) [file ppat.1007926.s007.tif]

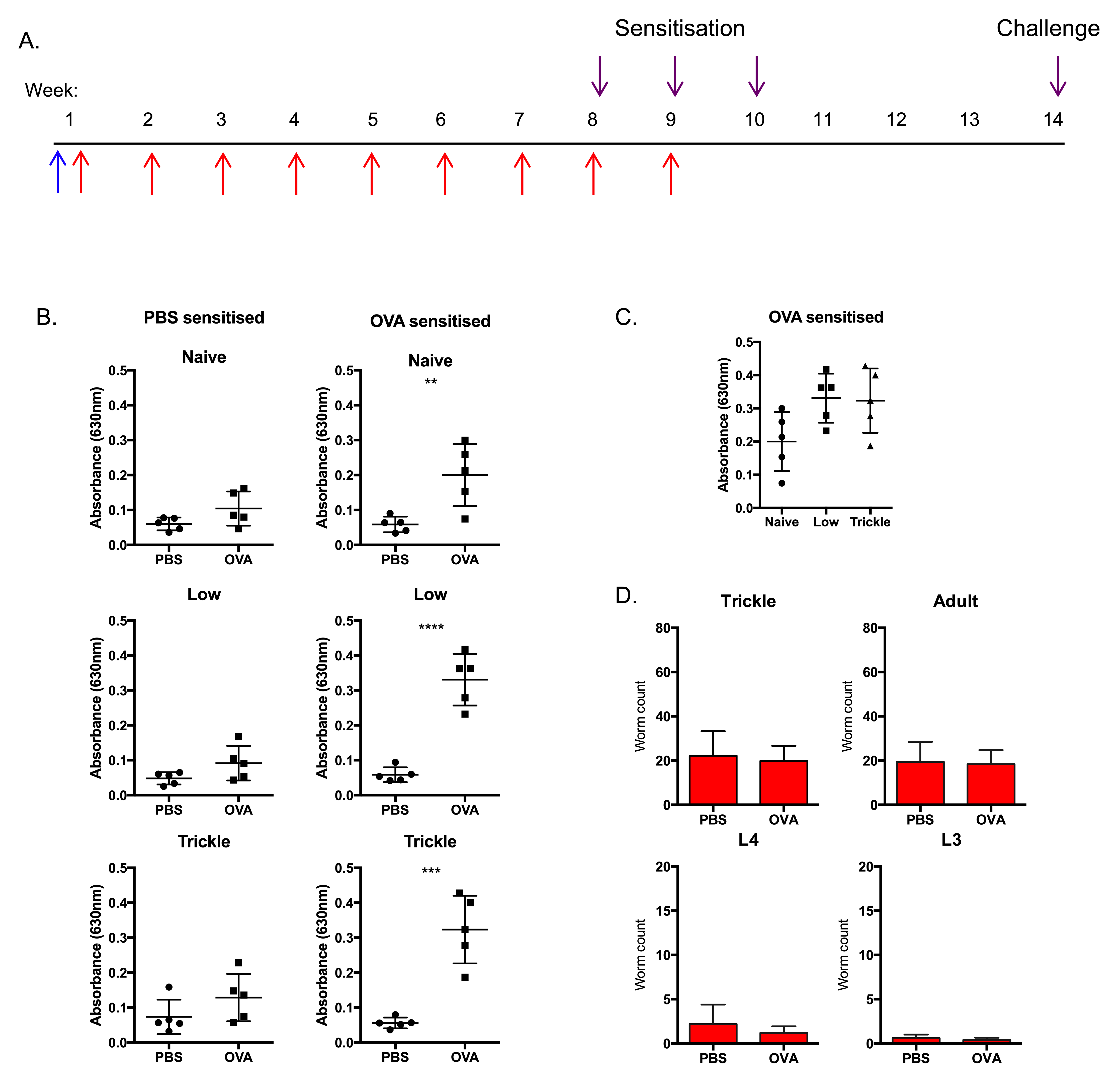

Supplement: S8 Fig — C57BL/6 mice were infected with a single low dose or trickled with T. muris over 9 weeks. At week 8 following the first infection mice were sensitized with 50 μg OVA antigen in 2mg of Alum or PBS control for 3 weeks. 4 weeks following the final sensitization all mice were challenged with 50 μg OVA by intradermal injection in the right ear and a PBS control injection in the left ear. A) Timeline of T. muris infection and sensitisation and challenge of OVA/PBS. B) Immediate hypersensitivity response in mice following PBS and OVA challenge. Hypersensitivity was determined by measuring skin permeability after OVA challenge using an Evans Blue assay. Levels of Evans Blue were quantified by absorbance and data are represented as arbitrary absorbance values at 602 nm. C) Side-by-side comparison of hypersensitivity response following OVA stimulation in naïve mice, low dose infected mice, and trickle infected mice from panel B. D) Worm burdens of T. muris trickle infected mice. Data presented as mean +/- SEM. Statistical test calculated by an unpaired t-test, ** = p<0.01, *** = p<0.001, **** = p< 0.0001, n = 5. (TIF) [file ppat.1007926.s008.tif]

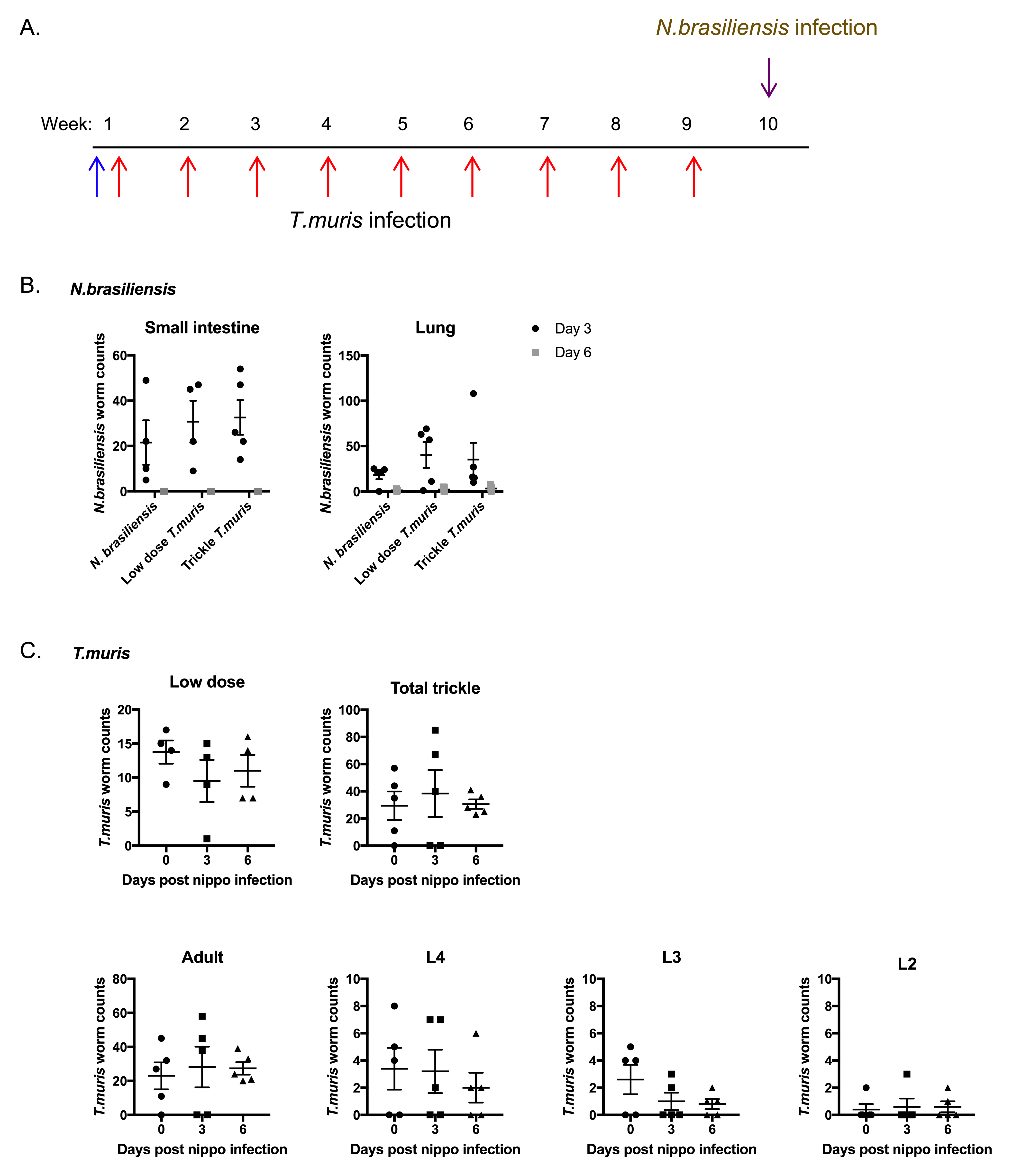

Supplement: S9 Fig — C57BL/6 mice were infected with a single T. muris low dose (20 eggs) or trickle infected of 9 weekly low doses by oral gavage. At week 10, T. muris infected mice and naive mice were infected with a single high dose (300 larvae) of N. brasiliensis by subcutaneous injection. At day 3 and day 6 following the N. brasiliensis infection, worm burdens of N. brasiliensis from the lung and small intestine and T. muris in the caecum, were counted. A) Timeline of infection regime. B) Worm burdens of N. brasiliensis in the small intestine and lung. C) T. muris worm burdens from low dose infected and trickle infected mice. Burdens of total worms and well as adult, L4, L3 and L2 were counted for trickle infected mice. Data presented as mean +/- SEM. Statistical analysis was carried out using a one-way ANOVA followed by post-hoc Tukey’s test. n = 5. (TIF) [file ppat.1007926.s009.tif]

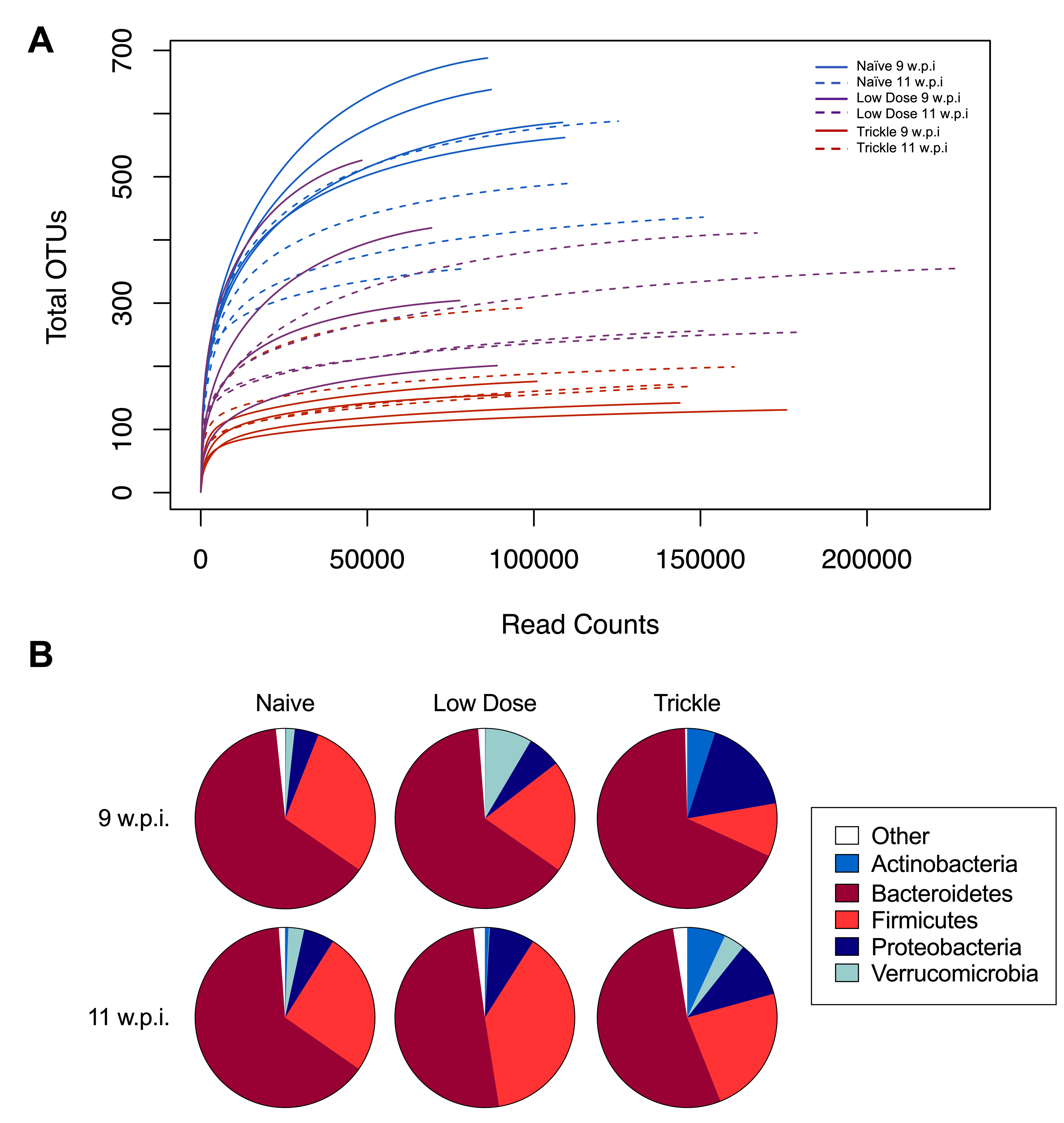

Supplement: S10 Fig — Following trickle infection mice were sacrificed at week 9 and week 11 post primary infection and the final stool microbiome was measured using 16S sequencing. (A) Rarefaction curves for individual samples calculated in R using the vegan package. (B) Phylum level comparisons between groups. Data represents the mean from 4 mice. Phyla representing, on average, less than 2% of the population were grouped into “other.” (TIF) [file ppat.1007926.s010.tif]
