# Supplementary material for: Trickle infection and immunity to Trichuris muris
Source: PLoS Pathog. 2019 Nov 15;15(11):e1007926. doi: 10.1371/journal.ppat.1007926 (PMC6881069; doi:10.1371/journal.ppat.1007926)
Supplement: Attachment — Submitted filename: Rebuttal letter Plos pathogens.docx [file ppat.1007926.s011.docx]

School of Biological Sciences

Lydia Becker Institute of Immunology and Inflammation

Wellcome Centre for Cell Matrix Research

Faculty of Biology, Medicine and Health

Manchester Academic Health Science Centre

A V Hill Building

The University of Manchester

Oxford Road

Manchester, UK

M13 9PT

30^th^ August 2019

Editors

Plos Pathogens

Dear Editors

Thank you very much for your email of 26^th^ July detailing the responses of the reviewers to our recently submitted manuscript **"Trickle infection and immunity to *Trichuris muris*" (PPATHOGENS-D-19-01054)**.

We appreciated the positive response of all the reviewers and also thank them for their insightful comments about our work. We have considered their comments carefully and have addressed all the points they raised below as follows:

**Part II – Major Issues: Key Experiments Required for Acceptance**

*Reviewer #1: There are 3 variables that change when the authors compare early time points versus the late time points – age of the mice, number of inoculations, and the amount of time that has passed since the first inoculation. Therefore, it is difficult to know what led to alterations in worm burden and Th responses at the later time points. What happens if they perform repetitive inoculations for the first 3-5 weeks and then examine at week 11?*

Response: We do not believe the age of the mice is a major influence here as all the mice in the current experiments would still be regarded as mature adults and not aged mice in any sense. Furthermore, our previous work on aged mice suggests that differences in immune responsiveness and resistance only become apparent in very old mice (>78 weeks old) and it is enhanced susceptibility and not enhanced resistance that we see in the aged mice (Humphreys *et al*, 2002, *Inf. Immun*, 70, 5148-5157). This is opposite to the increase in resistance which we actually see at week 11 after trickle infections.

The second issue raised is the time that has passed since the first inoculation. We would argue that it is not the *time period* that has elapsed between that is critical here. Extensive previous work has shown that the major influence upon the generation of resistance to

*T. muris* is ***dose*** of infection in the context of a particular genetic background (reviewed in Klementowicz *et al*, 2002, *Sem. Immunol.Immunopath*, 34, 815-828; Hurst *et al*, 2013, *Parasitology*, 140, 1325-1339; Grencis *et al*, 2014, *Immunol.Rev*, 260,183-203) ).

In all inbred strains of mouse tested to date a single low dose of eggs (less than 25 eggs) generates a strong Th1 response and the parasites progress to adulthood, patency and long-lived infection. These mice are very susceptible to a subsequent high or low dose challenge infection, even after anthelmintic treatment to clear the priming infection. In almost all inbred strains of mouse tested to date a single high dose of eggs (hundreds of eggs) generates a strong Th2 response and the parasites are expelled before they reach adulthood. The resistance is strong and complete to either a high or low dose challenge (Bancroft *et al*, 2001, Int. J. Parasitol. 31, 1627-1637).

The present data clearly shows that that vast majority of worms present after 3, 5 or 7 low dose infections are adults, L4 or L3 parasites (i.e. stages associated with immunomodulation and chronicity; Else *et al*, 1989, *Parasitology*, 98, 275-282) demonstrating worm numbers increase over this time period and there is no evidence of resistance. Analysis of a variety of immune parameters shows a dominant Th1 response at these time points. Taken together, there is no reason to believe that the chronic infections generated after just two or three low dose infections will subsequently result in worm loss if examined at 11 weeks as the response to the parasites is Th1 dominated and Th1 responses do not mediate protective immunity. Moreover, we now include a new dataset in which mice were given a three-week trickle infection and challenged at week 12 which shows the mice do not subsequently become resistant over time. This data is now in Supplementary Fig 4 (previously Fig 5) as we have moved this group of experimental data to the supplementary section as suggested by Reviewer 3 (below). Thus, we believe that it is the number of infection events which is the major driver of resistance here.

*Reviewer #2: As stated above, careful studies of this nature are extremely important and are likely to be very informative. Although the authors have nicely characterized the model and the dramatic changes that are occurring between weeks 9-11, there is no explanation of the mechanisms that drive this transition. In its current state, the study is very observational. There appears to be an important transition in the Th2 cells that occurs after week 9 despite the fact that by week 9 the mice have been challenged 7 times and have 50 worms. Why do 2 more challenges drive this difference, is it just a magnitude issue? What do the authors think is happening, considering that little change in the worm burden is occuring between weeks 7-9 it doesn’t seem like its simply antigen load. It would be great if they authors could determine whether T cells transferred on week 9 and 11 differed in their ability to promote immunity using a gain of function approach. They should them determine what has truly changes in those T cells to give mechanistic insight. Transcriptional profiling would be ideal to accomplish this assuming that its T cell intrinsic. In general, the manuscript needs more mechanistic data to be appropriate for publication.

Also, although interesting the microbiome analysis seems out of place and is very observational in its current form. What are the authors trying to say about these findings and how they relate to the development of immunity?*

Response: The reviewer raises a number of points.

The fact that much of the study is observational is true, but the CD4+T cell and ILC2 depletions are *in vivo* intervention studies and do address important mechanistic questions.

The reasons why one or two more challenges begin to make a difference in terms of resistance is of course a central question that we do not have a complete answer to. This is akin to asking why it takes so long for immunity to become evident in apparent in human populations etc. We think the current study does indeed help provide some clues to help answer this, at least in part.

Our data supports the notion that it takes multiple numbers of challenges, particularly by the earlier larval stages of the parasite to stimulate a strong enough Th2 response to prevent development of the later infections. We know from the literature, that the early pre-L3 stages of infection are the stages that promote resistance and later stages suppress resistance (references). The reason why this extra “couple” of low dose challenges enables this remains to be discovered and it may be that for different genetic backgrounds, more or fewer challenges are required. The data is supportive of the concept of a level of Th2 response must be reached that is sufficient to generate a protective anti-worm response. It is clear that this can be achieved with one very large bolus dose, but with low dose infections that are more representative of natural exposure multiple infections are required. It is noteworthy that despite much work over the years from many laboratories we still do not understand the detailed mechanistic basis of why the same inbred strain of mouse makes a Th2 response to a high dose of *T. muris* infection and a Th1 response to a low dose *T. muris* infection. Understanding this will undoubtedly inform on the question raised by this reviewer on the induction of protective responses during trickle infections. We believe tackling this problem is beyond the scope of the present study but certainly worthy of further investigation.

Although the CD4+ T cell/ ILC2 depletion experiments show that CD4+ T cells are key to the immunity generated by repeated trickle infections, we did not include experiments looking at adoptive cell transfer of immunity. We have carried out such experiments, but they are inconclusive. We adoptively transferred purified CD4+T cells from the mesenteric lymph node from mice trickled for 11 weeks, a time at which protective immunity is ongoing. The data from the experiment is shown below for the reviewer’s information (Figure 1). No differences in worm burdens were seen between experimental and control group. We further transferred the same cell population into immunodeficient recipients, but the mice became ill and had to be culled. We do not think inclusion of this data into the manuscript is informative. The reasons for the lack of transfer of immunity can be manifold; non-optimum time point of harvest or transfer relative to infection, too few cell numbers etc.. Also, as only partial immunity is evident following trickle, it is very likely that there are mixed populations of Th1/Th2 in the transferred populations (supported by parasite specific antibody subclass data, i.e. elevated IgG1 and IgG2a/c) which may be less effective upon transfer. Thus, the adoptive transfer approach will require a considerable further experimental investigation and dissection beyond the scope of the current manuscript. Thus, we believe that the CD4+ T cell depletion is strong data and robustly demonstrates their importance in resistance induced by trickle infection.

Figure 1 CD4+ T cell were isolated and purified from week 11 trickle infected mice. CD4+ T cells

were I.V. injected into C57BL/6 mice and received a single low dose (20 eggs) infection the following day. A) Worm burden was counted at day 35 p.i. B) ELISA to quantify *T. muris* specific IgG1 and IgG2a levels. Data presented as mean+/- SEM, n=5.

Also, the suggestion to transcriptionally profile the cells is interesting, but would require considerable extra work and as extensive work has already shown that immunity to *T. muris* does require Th2 cells it is possible that the level of information gained would be incremental, largely observational and not change the central conclusions of the present work. We accept that it is something that should be considered going forward but do not feel it is necessary to support the conclusions we make in the present paper.

The data on the microbiome analysis was included as we believe it adds important new information to our current understanding of the influence of helminth infection on the intestinal microbiota. Previous work (by us and others) has shown that a single low dose *T. muris* infection significantly changes the microbiota – although the changes seen are far more marked than those observed in studies of humans infected with *T. trichiura*. One obvious difference is the mode of helminth challenge and the work presented here presents data that is far more in accordance with the changes seen in human populations infected by whipworm, hence its inclusion. Again, this observation opens up a number of questions for future work that can examine the basis of the changes that occur as a different immune responses begins to build against the worm.

Reviewer #3: none

**Comments related to editoral changes:**

1*) many of the figure legends do not contain enough information to allow the reader to understand the experiment, this is especially true of the supplementary figures. For instance no information is given as to the length or timing of the Edu pulse (figures 4 and 7 or how antibody levels were quantified (Supplementary)*

We apologise for this and have been through the legends, especially in the supplementary section and added more information as requested.

*2) It would be useful for the authors to clearly state in the text that the microbiota analysis was performed in stool, and to discuss the drawbacks of using stool versus cecal contents and mucous layers in terms of exploring the true extent of the impact of infection.*

We have taken on board these comments and have added text to cover these points, highlighting that in the case of low dose *T. muris* infection, stool microbiota analysis reflects the caecal microbiota analysis quite well.

*3) I am not sure I agree with the authors conclusion that antibody levels were unaffected by CD4 T cell depletion (they seem to be decreased)? As no evidence regarding the role of antibodies is provided it would be better to refrain from raising their involvement as being unlikely and instead simply say that this was not determined.*

This point is well taken. CD4+ T cell depletion does have an effect albeit a small effect, i.e. significant levels of anti-parasite antibody are still evident after depletion. However, we cannot rule out that antibody did not play a role and thus we take the reviewer’s advice and have modified the text accordingly.

**Part III – Minor Issues: Editorial and Data Presentation Modifications**
Please use this section for editorial suggestions as well as relatively minor modifications of existing data that would enhance clarity.

*Reviewer #1: In Fig 5b, have the authors confirmed that the dose of anthelmintic used was effective at clearing worms?*

Yes, this was confirmed by examining the faeces for eggs. This information has now been added to the Figure legend.

*For Fig 4, please specify the time points and egg doses in the legend*

This has been done

Reviewer #2: (No Response)

*Reviewer #3: Comments related to data and figure legends:

1) The experimental design of the work presented Figure 5 was extremely interesting and is given a strong degree of relevance by the authors in that a entire subsection (lines 160--174) is detected to it. However a very limited data set is shown and as such the data stands in stark contrast to the rest of the manuscript. Many questions remain open including the impact on specific larval stages, the association with various cytokines and antibodies, epithelial cell responses and so forth.

For these reasons I would recommend one of the following options i) extending the dataset, ii) moving it to the supplementary information, or iii) removing this figure and instead working further to determine whether ILC2 or CD4 T cells were involved in the long-lasting protection observed, and to determine the functional significance of antibodies (perhaps by passive transfer exps) at this time.*

We agree that this is an important aspect of the work. However, we also agree that the dataset is relatively small and as we do not delve into the mechanistic basis for the memory – we have decided to take this reviewers suggestion (ii) and place the data in the supplementary section instead. We thank the reviewer for this useful suggestion and improvement to the manuscript.

*2) It is not clear whether the data shown in Figure 9 is pooled from two experiments or is one data set that is representative of 2 exps. Given the worm counts are very variable and the possible trend toward increased worm counts in the DTx treated mice it would be useful to either show the second experiment or to increase the total number of mice investigated to increase power.*

The data in figure 9 is pooled from two experiments. This is now clearly stated in the Figure legend. We agree with the reviewer that there is some variability in the dataset, however, we argue that in the Dtx treated mice the deviation from the mean of roughly 25 worms was not significant. Across multiple experiments (included in the manuscript) the mean number of worms present at week 11 in our trickle-infected mice was around 20-30. This is consistent with the notion that protective immunity develops against infection, given that at this time point mice had received a total of 180 eggs. RAG mice receiving the same treatment had a worm burden of roughly 80 worms/mouse providing a baseline for the number of expected parasites in mice in which protective immunity does not develop (Data present in Figure. 5). Therefore, we do not believe that expanding the current data to increase power would provide any novel information.

*3) The pictures of tuft cells shown in Fig8 are not convincing. Better representative pictures and/or pictures at higher magnification should be shown to convince the readers the authors performed a correct quantification of tuft cells. It might also be useful to show pictures from the SI of Nippo infected mice for comparison and again to convince the readers correct techniques were employed in enumerating tuft cells.*

We apologise for the picture quality – we have now corrected this. Also, to help convince this reviewer that we can enumerate tuft cells accurately we include a comparative example data from mice infected with *Hymenolepis microstoma* and *Heligmosomoides polygyrus*, intestinal helminths present in the small intestine that both generate a very strong intestinal tuft cell response for their information (Figure 2). We would propose that this is not required for inclusion in the manuscript as it is a methodological point rather than an issue that adds to the science of the paper.

Figure 2 Tuft cell staining in the small intestine following infection with H. microstoma and H. polygyrus. For H. microstoma the definitive tuft cell marker Dclk1 is shown in red, here we include costaining with UEA-I lectin (green) a marker that stains a subset of tuft cells (Bjerkness 2012 PMID:22185794) confirming the identity of our Dclk1+ve cells. For comparison tuft cells were also identified in mice infected with H. polygyrus, here Dclk1+ve cells are labelled green.

We believe that the modifications that we have made based upon the reviewers’ comments has improved the manuscript considerably and we thank them for their time and efforts.

We hope that the manuscript is now acceptable for publication in Plos Pathogens and look forward to hearing from you.

Yours sincerely

**Professor Richard Grencis, FRSB, MAE**

Bill Ford Chair in Cellular Immunology

Phone: +44 (0) 161 275 5238

www.manchester.ac.uk/lydia-becker-institute

www.wellcome-matrix.org

www.immunophenotyping.org
